# A Review on Synthesis, Structural, Flame Retardancy and Dielectric Properties of Hexasubstituted Cyclotriphosphazene

**DOI:** 10.3390/polym13172916

**Published:** 2021-08-30

**Authors:** Siti Nur Khalidah Usri, Zuhair Jamain, Mohamad Zul Hilmey Makmud

**Affiliations:** Sustainable Materials and Renewable Energy (SMRE) Research Group, Faculty of Science and Natural Resources, Universiti Malaysia Sabah (UMS), Kota Kinabalu 88400, Sabah, Malaysia; sitinurkhalidahusri07@gmail.com (S.N.K.U.); mzhilmey@ums.edu.my (M.Z.H.M.)

**Keywords:** cyclotriphosphazane, flame retardancy, dielectric properties

## Abstract

Hexachlorocyclotriphosphazene is a ring compound consisting of an alternating phosphorus and nitrogen atom with two chlorine substituents attached to the phosphorus atom. The six chlorine atoms attached to this cyclo compound can be substituted with any different nucleophile that leads to changes in different chemical and physical properties. The major topics that were investigated in this research are the flame retardancy and dielectric properties of cyclotriphosphazene compounds. Cyclotriphosphazene compounds have high potential to act as a flame retardant, and this compound consists of two active elements attributed to its high flame-retardant character. This compound also demonstrated good ability as a flame retardant due to its low toxicity and less smoke produced. In addition, cyclotriphosphazene compounds were also investigated for their dielectric properties. Cyclotriphosphazene has high potential in the electrical field since it has dielectric properties that can be widely studied in the investigation of any potential application. This review presented literature studies focused on recent research development and studies in the field of cyclotriphosphazene that focused on synthesis, structural, flame retardancy, and dielectric properties of hexachlorocyclotriphosphazene compounds.

## 1. Introduction

The discovery of the area of organic chemistry led to a new era of material science. In the early years, the cyclotriphosphazene compound was extensively explored and had a high impact on innovation in the chemistry field. The cyclotriphosphazene compound was first investigated in the mid-1950’s by Allock and his co-workers. To date, this compound has been used in many fields due to its fascinating properties [1,2], and this compound is still being explored to make it more useful in modern technology.

**Figure 1:**
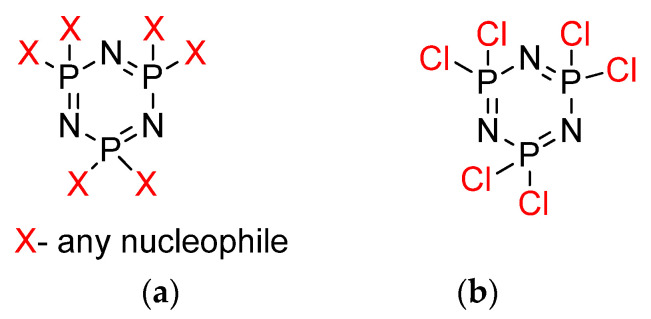
Chemical structure of (**a**) cycloytriphosphazene and (**b**) HCCP.

Cyclotriphosphazene (Figure 1a) is a ring compound that consists of alternating phosphorus and nitrogen atoms with two substituents attached to phosphorus atoms [3]. Cyclotriphosphazane, also known as hexachlorophosphazene, is a hexa-membered cyclic ring of organophosphazene with the molecular formula (NPCl_2_)_6_. This compound is easily modified by replacing the chloro atom existing on the phosphorus atom with an appropriate reagent [4]. Due to the high reactivity of the P–Cl bond, the corresponding substitution method allows for the introduction of a wide range of substituents [5] by nucleophilic reaction (Figure 1b) to obtain new cyclotriphosphazene derivatives having different chemical and physical properties depending on the characteristics of the substituted groups [6]. Many studies have focused attention on cyclotriphopshazene derivatives [7,8]. Previous research has been carried out by changing the side group -R of phosphazene, such as -NH_2_, -OR, -OC_6_H_5_, or fluorinated derivatives that are able to improve oxidative and thermal stabilities [9]. Furthermore, the properties of polyphosphazenes change as a result of the different side groups, such as elasticity, thermal stability, solvent resistance, biological utility, electro-optical properties, and so on, indicating that the polyphosphazenes can be adjusted and controlled depending on the materials they are applied with [10,11,12,13,14].

Dogan et al. also reported that cyclotriphosphazene derivatives containing a bis-aryl Schiff base having a different terminal group (H, F, Cl, and Br) have good thermal properties, which makes them suitable as a flame retardant additive to slow down the spread of the flame ignition or slow down the spread of flame after ignition of materials [15].

Cyclotriphosphazene compounds are extensively investigated, as they are reported to have potential in some industrial applications, such as in flame retardant [16,17]. Two active elements found in flame retardants are phosphorus and nitrogen, and these give a synergistic effect greater than a simple addictive. Their flame retardant effect combines good flame resistance and self-extinguishing ability in the polymer [18,19,20,21]. These materials also offer exciting technological prospects, as they can be applied as starting materials to synthesize industrially valuable substituted cyclotriphosphazenes. The derivatives of cyclotriphosphazene have been used as an additive in various fields such as special rubber [22] and biomedicine [23], the most common being in flame retardant materials [24].

Several cyclotriphosphazene derivatives with excellent properties have been synthesized by replacing Cl atoms of HCCP [25,26]. Before this, halogen was used as a flame retardant substance, but it is reported that these halides release toxic and corrosive substances during their decomposition and cause environmental problems [27]. Therefore, it is necessary to develop halogen-free and environment-friendly flame retardants to improve flame retardancy. Thus, cyclotriphosphazene compounds that contain phosphorus atoms as the main compound have shown high potential to replace halogen as an environmentally friendly flame retardant due to this compound possessing excellent properties such as low smoke emission, low toxicity, and the formation of a stable carbonized layer after burning [28].

Furthermore, related research constitutes a relatively new area that has emerged from the flame retardancy of cyclotriphosphazene to studies in the dielectric properties of this compound. Dielectric materials are non-conductors of electricity or can be defined as electrical insulators that can be highly polarized by an electrical field that is the material’s dielectric constant. Dielectric materials are non-conductors of electricity that can be substantially polarized by an electrical field, which is the material’s dielectric constant. On removal of the electric field, the material returns to its original state, and the time taken to do this is referred to as the relaxation period, which is a characteristic of the dielectric material.

In addition to cyclotriphosphazene and their dielectric properties, polymer compound are well known to be used in electrical applications, especially as an insulator, due to its properties of the superior service [29]. Thus, the cyclotriphosphazene compound is the most potent compound that can be used in advanced electrical devices. It has good flame retardant properties, which are also important in the build-up of the electronic application, and has a dielectric property. To conclude, an extensive study about these two related properties with high-potential compounds, which is cyclotriphosphazene, needs to be carried out to enhance multifunctional properties that might be beneficial in future production in an electrical field. 

This article presented a review of the recent synthesis of hexasubstituted cyclotriphosphazene compounds. The recent studies related to the flame retardancy and dielectric properties of this compound were also discussed. The paper is divided into four significant sections related to the synthesis and properties of cyclotriphosphazene compounds. The first section deals with the study of synthesis hexasubstituted cyclotriphosphazene, and its flame retardancy properties in recent years. The second section discusses the effect of the structural cyclotriphosphazene compounds, including the linking unit and enhancements achieved through the linking unit of the new hexasubstituted compounds. The third section deals with the dielectric properties of cyclotriphosphazene compounds and, finally, the fourth section details the combination of two properties which are flame retardancy and dielectric properties of cyclotriphosphazene that can be used as a reference since this compound has high potential in a high flame-resistant electrical application or other electrical applications, such as insulation, or as a capacitor in an electrical component responsible for the energy-storage properties of the devices. In addition, the use of dielectric materials in sensors for a multitude of applications, such as self-driving cars, has made dielectric science and technology research even more significant than before.

## 2. Synthesis of Hexasubstituted Cyclotriphosphazene and Flame Retardant Properties

Various approaches are available for cyclotriphosphazene synthesis methods. The starting material to synthesize this compound is hexachlorocyclotriphosphazene (HCCP). An important task in preparing hexasubstituted cyclotriphosphazene is to react HCCP with other compounds to form a new hexasubstituted cyclotriphosphazene, where the chlorine atoms in HCCP are fully substituted with a new nucleophile to form a halogen-free cyclotriphosphazene compound. Over time, extensive literature has developed on cyclotriphosphazene as a flame retardant. Incorporation of HCCP into compounds increases the resistance of the material towards ignition. The flame retardant function minimizes the flame risk and prevents a small fire from becoming a major catastrophe [30]. Flame-resistant materials play a critical role in preventing property damage. The removal of heat from the materials that can burn and the creation of char during the fire results in flame retardation, interrupting the contact from combustion. In addition, some studies reported cyclotriphosphazene with an alkyl chain increases the thermal properties and flame retardancy because of phosphorus and nitrogen-flame-retardant synergy [17]. Recently, a number of novel synthesis approaches have been proposed to synthesize a new hexasubstituted cyclotriphosphazene with flame retardancy properties. In this work, different synthesis approaches were reviewed to synthesize a cyclotriphosphazene compound with flame retardancy properties. It provided an up-to-date summary of the synthesis method and also the flame retardancy of the compound. Limiting Oxygen Index (LOI) is a common technique used to evaluate the flame retardancy of textile materials and films. Materials exhibiting LOI values of 25% and greater are considered self-extinguishing. Usually, inherently flame retardant materials exhibit LOI values greater than 30%. Generally, UL-94 rating tests are widely used for the flammability of plastic standards, which are used to evaluate the material’s ability to extinguish after being ignited [31,32]. In particular, the horizontal or vertical sample is ignited numerous times according to a specific flame height and flame application angle, and the evaluation is based on the sample’s ignition duration and burning performance. The UL-94 rating performance of polymers can be multiply appraised into many distinct levels depending on the burning speed, burning time, anti-dripping ability, and if the bead is burning [33]. Table 1 summarizes the studies on the synthesis of hexasubstituted cyclotriphosphazene and their flame retardancy properties.

## 3. Effect of Linking Unit in Flame Retardancy Properties of Cyclotriphosphazene

**Figure 2:**
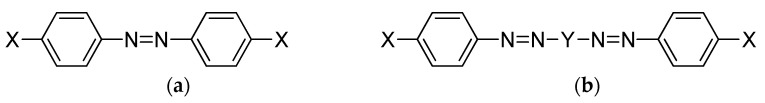
General chemical structure of (**a**) the monoazo linking unit and (**b**) the bisazo linking unit.

Linking units are usually structural units that connect one core to another, which maintain the linearity of the core while being compatible with the rest of the structure [3]. As the connecting units give a point of link up in synthesis, compounds with linking groups are more accessible to synthesis than those with direct bonds. There are several types of linking units, which are azo (-N=N-), Schiff base (-CH=N-), acetylene (-C≡C-), diacetylene (-C≡C-C≡C-), and stilbene (-CH=CH-). Based on the reports from previous research, a comparison of two linking units, which are Schiff base and azo linking units, has been made. This study concluded that Schiff base linkage showed higher thermal stability than the azo due to rigidity in the Schiff base’s linkage [68]. Furthermore, strong molecular interaction in Schiff base derivatives results in high temperature compared with azo derivatives. Until now, few linking units have been explored related to the enhancement of properties of the cyclotriphosphazene compound, which are the azo and Schiff base linking units. Generally, the azo linking unit can be classified into two types which are monoazo and bisazo linking unit (Figure 2), while the Schiff base functional group consists of a carbon-nitrogen double bond (C=N) with the nitrogen atom connected to the aryl or alkyl group but not with the hydrogen. The general formula of Schiff base is R_1_R_2_C=NR_3_ with R as a side chain [69]. The Schiff base is an intriguing linking unit that provides a stepped-core structure that allows molecules to maintain linearity, provide excellent stability, and change the physical properties contributed by the linking unit itself [70].

**Figure 3:**
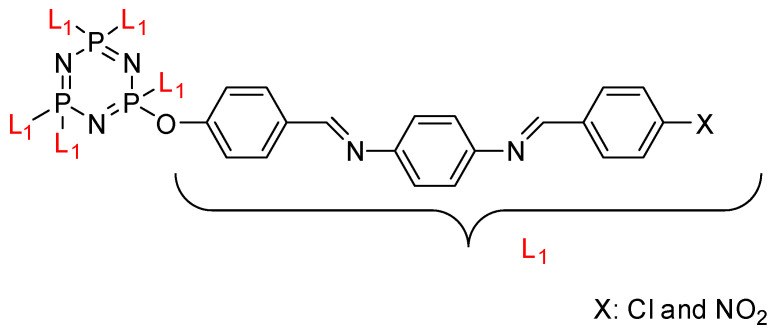
Hexasubstituted cyclotriphosphazene with two Schiff base linking units.

Some authors have driven the further development of studies in linking unit effects in flame retardancy as the contribution of several types of linking units in the enhancement of flame retardancy properties in cyclotriphosphazene compounds. Recently, Jamain et al. worked on synthesizing a new hexasubstituted cyclotriphosphazene compound with two Schiff base linking units and containing different terminal substituents as shown in Figure 3 [71]. The Schiff base linking unit was also reported to improve the flame retardant properties due to its thermal stability [72]. This research aimed to study the flame retardant properties of hexasubstitutd cyclotriphosphazene compounds containing two Schiff base linking units using the limiting oxygen index (LOI). All the samples were prepared with 1 wt.%, and polyester resin was used as molding. The result obtained from this study reported that the LOI value increased from 22.53% of pure polyester resin to 24.71% when hexasubstituted cyclotriphosphazene compound was incorporated with polyester resin. The best results achieved from this study were from the compounds with nitro and chloro terminal groups with LOI values of 28.37% and 27.90%, respectively. The phenomenon was due to the electron-withdrawing properties of both nitro and chlorine groups, which induced flame retardancy properties. 

Other linking units were studied, azo and amide linking units, and their contribution to the flame retardancy properties of cyclotriphosphazene is summarized in Table 2. 

## 4. Dielectric Properties in Cyclotriphosphazene

A non-metallic substance with high specific resistance, a negative temperature coefficient of resistance, and a substantial insulating resistance is referred as a dielectric material. The dielectric material can also be defined as a non-conducting material that stores electrical charges. When a dielectric is placed in an electric field, the electric charges do not flow through the materials. Electric charges slightly shift from their average equilibrium positions, causing dielectric polarization. Positive charges flow in the direction of the field, while negative charges shift in the opposite direction of the field due to dielectric polarization. This phenomenon yields an internal electric field, which in turn reduces the overall electric field within the dielectric materials.

Cyclotriphosphazene compounds and their versatility of substituent placed in cyclic and macromolecules backbones make this compound have significant potential in applications such as dielectric [77,78,79], additives of flame retardant [26,80], fluorescence materials [81,82], and liquid crystals [83]. Recently, there have been many studies reported related to the dielectric properties of cyclotriphosphazene compounds. Dielectric properties such as dielectric loss, dielectric constant, and conductivity are the most common properties extensively studied to evaluate solid materials. In addition, dielectric constant measurements are among the most popular methods of evaluating solid materials such as electric insulators and polymers, in which dielectric constant measurements can be performed easier than with chemical analysis techniques. Impedance spectroscopy is a relatively recent and powerful technique for determining several electrical properties of electrolyte materials and their interactions with electronically conducting electrodes. The permittivity of a material describes its ability to absorb, transmit, and reflect electromagnetic energy. These also are important properties required to design electronic devices and have numerous potential application areas in multifunctional electronic and optoelectronic devices [84,85,86,87].

Koran et al. recently worked on synthesizing cyclotriphosphazene derivatives from the reaction of substituted chalcone compounds containing different organic side groups at the para position with cyclotriphosphazene containing bearing dioxypheneyl [88]. Dielectric properties of the final compound, which is the dielectric constant, were measured using an impedance analyzer. From this study, the result obtained reported that the cyclotriphosphazene compound containing methoxy was observed to have a high dielectric constant. This is due to the increase in polarization with a conjunction. Meanwhile, compounds containing methyl groups showed the lowest dielectric constant. Thus, compounds containing methyl groups were chosen to determine the influence of Eu3+ doping on the dielectric properties of phosphazene. In addition, the dielectric behavior of new oxime-cyclotriphopshazene derivatives was also explored [79]. Cyclotriphosphazene compounds bearing oxime ether and ester as side groups were synthesized in this study (Table 3). The dielectric properties, which are dielectric loss and dielectric constant of this compound, were measured using an impedance analyzer. The results reported that decreases in the dielectric constants were obvious at low-frequency values. On the other hand, when closing the higher-frequency levels, the decline of the dielectric constants was reduced. The reason behind the results is the existence of polar groups on the structure, which lead to an increased dielectric constant. The carbonyl group which is bonded to oxime-ester groups made the dielectric constant higher compared with other derivatives. Both studies also reported that phosphazene derivatives are promising candidate materials in multifunctional and optoelectronic devices.

In addition, there are studies that reported on the development of phosphazene imine-modified epoxy composited for low dielectric. One study aimed to synthesize the phosphazene imine (PZ-imine), and the dielectric properties of this compound were measured [89]. Based on the result obtained, the dielectric constant for neat epoxy were 1, 3, 5, and 7 wt.% of PZ-imine, reinforced epoxy nanocomposites were 3.42, 3.10, 2.93, 2.75, and 2.41, respectively, the dielectric loss of neat epoxy were 1, 3, 5, and 7 wt.% of PZ-imine, and reinforced epoxy nanocomposites were 0.25, 0.20, 0.18, 0.14, and 0.12 at 1 MHz, respectively. When the frequency increases with a decrease in the orientation of polarization and its dipole moments need a longer time than the electronic and ionic polarization, this decreased the dielectric constant. In this regard, the addition of PZ-imine to the epoxy helped the reduction in the dielectric constant and dielectric loss values. In modified epoxy resins, the bulky group of PZ-imine efficiently inhibits dipole orientation and relaxation. The PZ groups minimize the inter-phase contact between the inorganic reinforcement and the organic domain, significantly lowering the dielectric constant of the resultant nanocomposites [90]. The influencing action of PZ causes a drop in the dielectric constant value due to an increased free volume to some extent. The dielectric loss of PZ-imine nanocomposites reduces as the amount of PZ-imine in the epoxy matrix increases. Direct current conduction, space charge migration, and the movement of molecular dipoles are all phenomena that contribute to the value of dielectric loss in materials. Polymer reinforced with PZ-imine results in an insulating layer outside the dielectric cores that control the migration and build-up of space charges within the nanocomposites, resulting in a lower dielectric loss. 

The information provided in Table 4 reveals that hexasubstituted cyclotriphosphazene was studied for its dielectric properties. It has fascinating properties that may contribute to the electrical field and details of explanation on how the structure affects the properties. 

## 5. Flame Retardant and Dielectric Properties of Cyclotriphosphazene

The research on cyclotriphosphazene compounds usually only focuses on one property, either flame retardant or dielectric properties. Regarding the development of cyclotriphosphazene, a few articles were published related to studies of a combination of two properties: flame retardant and dielectric properties on cyclotriphosphazene. 

**Figure 4:**
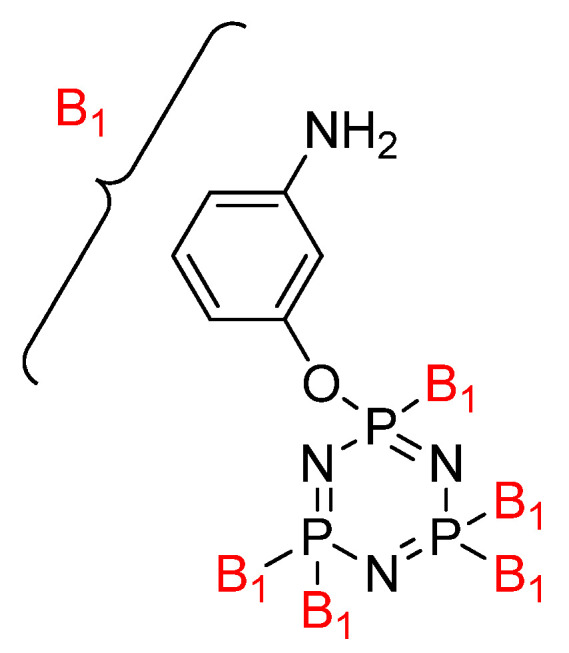
Structure of hexa(aminophenyl)cyclotriphosphazene.

Krishbaderi conducted an experiment to synthesize the hexa(aminophenyl)cyclotriphosphazene-modified cyanate ester composite for high temperatures as illustrated in Figure 4 [96]. This study aimed to enhance CEs thermal stability and flame-retardant properties since CE has excellent thermomechanical properties that make it useful in many microelectronic applications. Hexa(aminophenyl)cyclotriphosphazene was synthesized from the reaction between hexachlorocyclotriphosphazene with a mixture of 4-acetamidophenol and calcium carbonate. The CPA/CE composite was prepared, and the flame retardancy and dielectric properties of this compound were measured using the Limiting Oxygen Index (LOI) and an impedance analyzer.

The results obtained showed LOI values of 5%, 10%, and 15% and CPA/CE were 38%, 41%, and 44%, respectively. It showed that the LOI value improved with the increase of both phosphorus and nitrogen content. For the dielectric properties measured, the CPA/CE dielectric constant and dielectric loss were lower than the neat CE matrix. It also concluded that it is quite apparent that incorporating units with low polarity and large molar volume is essential for any molecule to have a low dielectric constant. Data obtained also indicate that CE containing cyclotriphosphazene can be found in micro-electrical applications since it has good thermal properties, flame retardancy, and dielectric behavior. 

In addition, Lin and Chang also studied the development of flame retardancy and dielectric properties of cyclotriphosphazene [97]. Their study aimed to synthesize a thermally stable, flame-retardant, and dielectric polymer from cyclotriphosphazene compounds. The hexachlorocyclotriphosphazene was synthesized to form cyclotriphosphazene containing acetylene, phenyl phenoxy, and also cyclotriphosphazene-containing styrene. The LOI and dielectric properties were referred to as the dielectric constant and dielectric losses of these three compounds were determined. From this study, the hexachlorocyclotriphosphazene compound with phenyl phenoxy had a high LOI value and char formation percentage, while for dielectric properties test, the cyclotriphosphazene compound containing styrene showed the higher dielectric properties with 2.40 of dielectric constant and 0.0014 at 1gHz of dielectric losses, respectively. This compound showed excellent dielectric properties and may be useful as a future low dielectric. 

There is a study reported on the synthesis of halogen free flame-retardant cyclotriphosphazene nanofibre-reinforced polybenzoxazine/epoxy [98]. Flame retardancy, thermal stability, and dielectric properties (dielectrics constant and dielectric loss) of this compound were measured in this study. The flame retardancy of this compound was measured using LOI and UL-94. Based on the result obtained, the LOI value of this compound increased when the concentration of compound increased, and 0.5, 1.0, and 1.5 wt.% showed 37%, 38%, and 41%, respectively. For dielectric properties, the dielectric constant showed 4.4, 4.0, and 3.4 with the increase of the PZT-fiber loading level of 0.5, 1.0, and 1.5 wt.%, respectively, for 1MHz at 30 degrees. These results indicated that when temperature increases, the dielectric constant will reduce significantly. The dielectric loss will reduce significantly with the increasing temperature, and the reverse trend in this case of dielectric loss was observed at 170. This study also reported that nanocomposites-based PZT fiber inhibits the lower value of the dielectric constant and dielectric loss over a wide range of temperatures, indicating its suitability for effective and thermally stable, flame-retardant dielectric material for micro electric applications.

Aromatic polyimides are usually used as a matrix material in printed circuit boards. When conventional polyimides are exposed to UV, they suffer from severe thermal breakdown due to internal heat generation [99]. Continuous usage and continuous exposure to high energy radiation even cause fire to the host materials. Namely, flame-retardant compounds such as phosphorus and halogen insertion provide heat resistance and preserve the host matrix materials. However, there are few publications available on polymer matrix precursors based on phosphorus and halogen compounds. It was recently discovered that when phosphorus and nitrogen-containing precursors are reinforced or co-polymerized, they improve the host polymers’ thermal performance and flame retardancy [100,101,102]. Revathi et al. worked to develop phosphazene-core-based polyamide and polyhedral oligomeric silsesquioxane (POSS)-reinforced phosphazene polyamide nanocomposites [103]. This study succeeded in developing new POSS, and cyclotriphosphazene nanocomposites with improved thermal stability, antibacterial activity, and low dielectric constants may find high-performance applications in coatings and microelectronics. This polyamide was prepared in three steps, which were the synthesis of hexakis (4-acetomidophenoxy) cyclotriphosphazene, synthesis of hexa (aminophenol) cyclotriphosphazene (PZI), and also the preparation of a neat PZI matrix and POSS/PZI nanocomposites. The developed polyamide and its composites were studied for optical, thermal, and dielectric properties. From the result obtained, the 10 wt.% of POSS/PZI showed higher flame retardancy. This is due to the silica moiety in addition to the flame retardancy of phosphorus and nitrogen atoms. For dielectric behavior, the dielectric constant was measured using an impedance analyzer in the frequency range 100 Hz to 1Mhz. From the result obtained, it was observed that the higher percentage reinforcement of POSS (10 wt.%) into the PZI matrix showed a lower value of the dielectric constant (k = 2.1).

**Figure 5:**
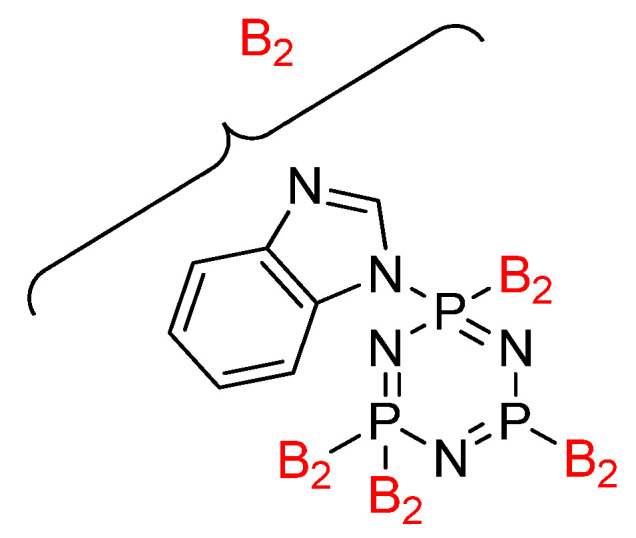
Benzimidazoyl substituted cyclotriphosphazene (BICP).

In addition, a seminal contribution was made by Cheng et al. in studying the hexasubstituted cyclotriphopshazene compound and its potential properties [49]. This study focused on the synthesized benzimidazoyl-substituted cyclotriphosphazene (BICP) (Figure 5) as a latent flame-retardant curing agent for one-component epoxy resin systems with excellent comprehensive performance. The flame retardancy, dielectric, and also mechanical properties of these compounds were also studied. From the result obtained, the LOI value of these compounds was recorded highest with the value of 33.5%, and the UL-94 test achieved a V-0 rating. This indicated that the BIMP enhanced the flame-retardant properties of epoxy resin without altering other properties when BICP cured EP. For the dielectric properties, the comparison of EP/BICP and EP/BIM was performed to indicate which of these two combinations have potential in the electric and electronic fields. This comparison concluded that EP/BICP has a lower dielectric loss and constant compared with the EP/BIM. The result of this research found clear support for increasing the free volume and/or decreasing the polarization of the materials, and the dielectric constant can be lowered. As a result, it was determined that the lower dielectric constant of EP/BICP thermosets could be attributed to the increased free volume of the EP matrix due to the integration of bulky, inflexible BICP molecules [104]. This simple strategy for preparing one-component flame retardant of epoxy resin with many potential properties could be useful in many fields. 

Table 5 reveals the structural contribution of the hexasubstituted cyclotriphosphazene compound that enhances the flame-retardant and dielectric properties. 

## 6. Conclusions

The hexasubstituted cyclotriphosphazene compound revealed that this compound is an excellent compound with fascinating properties, and it has been extensively studied as addictive for flame retardant. This is due to the presence of phosphorus and nitrogen atoms in this compound and the contribution of the linking unit and side chain that helps enhance the flame retardancy of this compound. In addition, hexasubstituted cyclotriphosphazene compounds also have their place in the electrical field, since the dielectric properties of these compounds are starting to receive attention from researchers. This compound has promising potential in the electrical field since it has flame-retardant and dielectric properties that could be valuable in manufacturing electronic devices.

## Figures and Tables

**Table 1:** Summary of the synthesis of hexasubstituted cyclotriphosphazene and their flame retardancy properties.

Title of Journal	Structure and Name of Compound	Flame Retardant Properties	Ref.
Flame retardant properties of cyclotriphosphazene derivatives for ABS	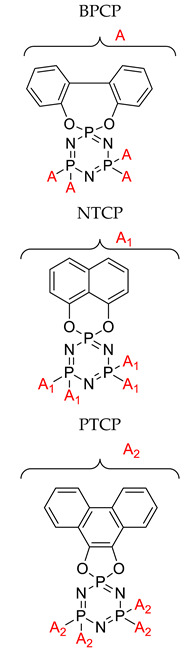	- LOI value: BPCP (21.2%, 22.1%, and 22.73% respectively), NTCP (22.3%, 23.1%, and 24.5% respectively, PTCP (23.1%, 24.1%, and 25.2%, respectively) - Additive used: 10 wt%, 15 wt%, and 20 wt% of each of BPCP, NTCP, and PTCP, respectively - Molding: ABS resin UL-94: Achieved V-1 rating	[34]
The flame retardancy and thermal stability properties of the poly(ethylene terephthalate)/hexakis(4-nitrophenoxy) cyclotriphosphazene system.	Hexakis(4-nitrophenoxy) cyclotriphosphazene (HNCP) 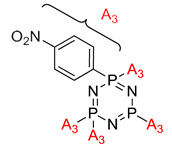	- LOI value: 35.1% - Additive used: 10 wt% of HNCP - Molding: Polyethylene terephthalate) PET UL-94: Achieved V-0 rating	[35]
Preparation and properties of halogen free flame-retardant blending modification polyester	Hexa(phenylamino)cyclotriphosphazene (HPACP) 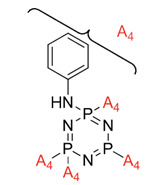	- LOI value: 28.3% - Additive used: 10 wt% of HPACP - Molding: Poly(ethylene terephthalate) PET UL-94: Not mentioned in this study	[36]
Synthesis of hexa-allylamino cyclotriphosphazene as a reactive flame retardant for unsaturated polyester.	Hexa-Allylamino Cyclotriphosphazene (HAC) 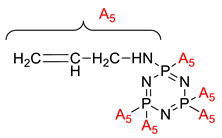	- LOI value: 36.5% - Additive used: Hexa-Allylamino Cyclotriphosphazene (HAC) - Molding: Diglycidyl ether bisphenol-A (DGEBA) UL-94: Not mentioned in this study	[37]
Synthesis of a phosphorus/nitrogen-containing compound based on maleimide and cyclotriphosphazene and its flame-retardant mechanism on epoxy resin.	Hexa(4-maleimido-phenoxyl)-cyclotriphosphazene (HMCP) 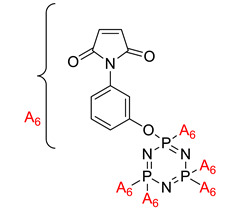	- LOI value: 36.5% - Additive used: Hexa(4-maleimido-phenoxyl)-cyclotriphosphazene (HMCP) - Molding: Epoxy Resin UL-94: Achieved V-0 rating	[38]
Synthesis of a novel flame retardant based on cyclotriphosphazene and DOPO groups and its application in epoxy resins	Hexa-[4-p-hydroxyanilino-phosphaphenanthrene-methyl)phenoxyl]-cyclotriphosphazene (HPMPC) 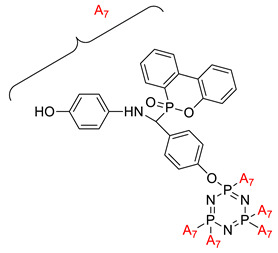	- LOI value: 36.6% - Additive used: 10.6 wt% of HPMPC - Molding: Diglycidyl ether bisphenol-A (DGEBA) UL-94: Achieved V-0 rating	[39]
Preparation of hexakis (4-aldehyde phenoxy) cyclotriphosphazene grafted kalonite and its synergistic fire resistance in poly(butylene succinate)	Hexakis (4-aldehyde phenoxy) cyclotriphosphazene (HAPC) 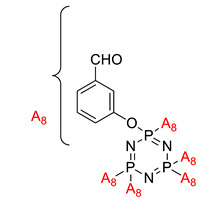	- LOI value: 40.3% - Additive used: 3 wt% of HAPC - Molding: Poly(butylene succinate) UL-94: Achieved V-0 rating	[40]
Synthesis, mechanical properties, and fire behavior of rigid polyurethane foam with a reactive flame retardant containing phosphazene and phosphate.	Hexa-(phosphite-hydroxyl-methyl-phenoxyl)-cyclotriphosphazene (HPHPCP) 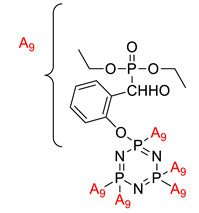	- LOI value: 25% - Additive used: 15 wt% of HPHPCP - Molding: Rigid polyurethane foams (FR-RFUFs) UL-94: Achieved HF-1 rating	[20]
Synthesis and characterization of flame-retardant rigid polyurethane foam based on reactive flame retardant containing phosphazene and cyclophosphonate	Hexa-(5,5-dimethyl-1,3,2-dioxaphosphinane-hydroxyl-methyl-phenoxyl-cycyclotriphosphazene (HDPCP) 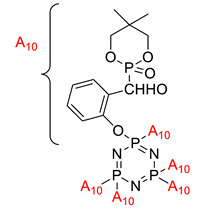	- LOI value: 25% - Additive used: 25 wt% of HDPCP - Molding: Rigid polyurethane foams (FR-RFUFs) UL-94: Not mentioned in this study	[41]
Application of cyclophosphazene derivatives as flame retardant for ABS	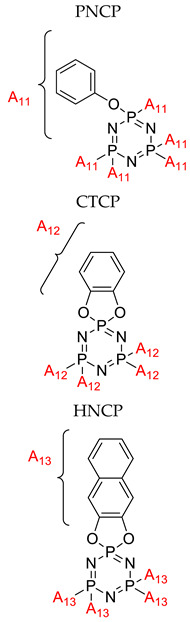	- LOI value: PNCP (20.8%, 21.5% and 22.1%, respectively) - CTCP (22.0%, 22.7% and 23.1%, respectively) - HNCP (22.9%, 23.6% and 24.9%, respectively) - Additive used: 10 wt%, 15 wt% and 20 wt% of PNCP, CTCP and HNCP, respectively - Molding: ABS resin UL-94: 15 wt% of the flame retardants were classified as the V-2 class, and 20 wt% of the flame retardants using PNCP were classified as V-2 but using HNCP or CTCP were classed as V-1	[42]
The non-halogen flame retardant epoxy resin based on novel compound with phosphenanthrene and cyclotriphosphazene double functional groups	Hexa-(phosphaphenanthrene-hydroxyl-methyl-phenoxyl)-cyclotriphosphazene (HAP-DOPO) 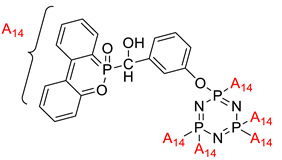	- LOI value: 35.2% - Additive used: 1.5 wt% of HAP-DOPO - Molding: epoxy resin UL-94: Achieved V-0 rating	[43]
Computer simulation study on the compatibility of cyclotriphosphazene containing the aminopropylsilicone functional group in flame retardant polypropylene/ammonium polyphosphate composites	APESP 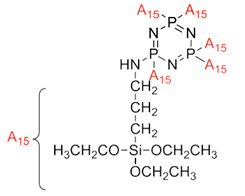	- LOI value: 26.5% - Additive used: APESP - Molding: Polypropylene (PP)/ammonium polyphospate (APP) UL-94: Achieved rating V-2	[44]
Hexa (eugenol) cyclotriphosphazene modified bismaleimide resins with unique thermal stability and flame retardancy	Hexa(eugenol)cyclotriphosphazene (HEC) 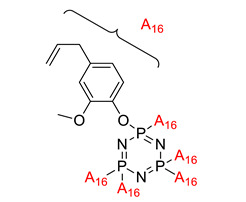	- LOI value: 39%, 48.4%, 50.1%, 49.8%, and 48.9%, respectively. - Additive used: Hexa(eugenol)cyclotriphosphazene (HEC) - Molding: 4,4′-bismaleimidodiphenylmethane (BMI) UL-94: Achieved rating V-0	[45]
Aminobenzothiazole-substituted cyclotriphosphazene derivatives as reactive fire retardant for epoxy resin.	Aminobenzothiazole-substituted cyclotriphosphazene derivative (ABCP) 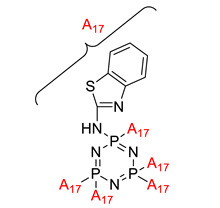	- LOI value: 31.2% - Additive used: Aminobenzothiazole-substituted cyclotriphosphazene (ABCP) - Molding: Epoxy Resin UL-94: Achieved V-0 rating	[46]
Effect of cyclotriphosphazene-based curing agents on the fire retardant of epoxy resin	Hexacyclohexylamino-cyclotriphosphazene (HCACTP) 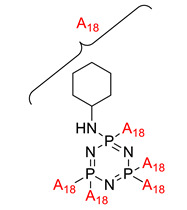 Diaminotetracyclohexylamino-cyclotriphosphazene (DTCATP) 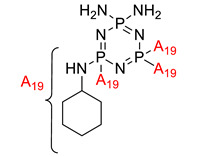	- LOI value: 25.3% and 22.2% respectively. - Additive used: Hexacyclohexylamino-cyclotriphosphazene (HCACTP) and Diaminotetracyclohexylamino-cyclotriphosphazene (DTCATP). - Molding: Epoxy Resin UL-94: Not mentioned in this study	[47]
The flame retardant properties and mechanism of poly(ethylene terephalate)/hexakis (para-allyloxyphenoxy) cyclotriphosphazene systems.	Para-allyl ether phenol derivative of cyclophosphazene (PACP) 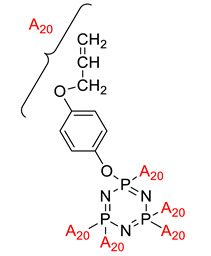	- LOI value: 33.5%, 33%, and 32.1%, respectively. - Additive: 5 wt%, 10 wt% and 15 wt% of PACP - Molding: Poy(ethylene terephalate) PET. UL-94: Achieved V-0 rating	[48]
Benzimidazolyl-substituted cyclotriphosphazene derivatives as a latent flame-retardant curing agent for a one-component epoxy resin system with excellent comprehensive performance.	Benzimidazolyl- substituted cyclotriphosphazene (BICP) 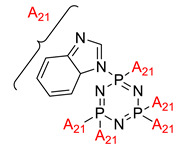	- LOI value: 33.5% - Additive: Benzimidazolyl-substituted cyclotriphosphazene (BICP) - Molding: Epoxy Resin UL-94: Achieved V-0 rating	[49]
Synthesis of melamine-cyclotriphosphazene derivatives and its application as flame retardant on cotton gauze	Melamine-Cyclotriphosphazene (MCP). 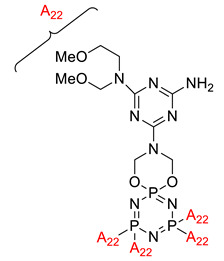	- LOI value: 16.9%, 23.5%, 24.6%, 25.4% 26.5%, and 25.8% respectively - Additive: Melamine-Cyclotriphosphazene (MCP). - Molding: Cotton Gauze UL-94: Not mentioned in this study	[50]
The flame retardancy and thermal stability properties of flame-retardant epoxy resin based on a-hydroxyphosponate cyclotriphosphazene	a-hydroxyphosponate cyclotriphos-phazene 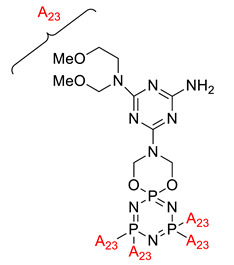	- LOI value: 30.7% - Additive: a hydroxyphosponate cyclotriphosphazene - Molding: Epoxy Resin UL-94: V-0 rating	[51]
Synthesis and flame retardant properties of cyclotriphosphazene derivatives containing Boron.	Cyclotriphosphazene derivatives containing Boron (CP-6B) 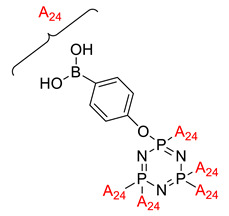	- LOI value: 32.3% - Additive: CP-6B - Molding: Epoxy Resin UL-94: Achieved V-0 rating.	[52]
An effective flame retardant for Poly(ethylene terephthalate) synthesis by phosphaphenanthrene and cyclotriphosphazene.	DOPO-TPN 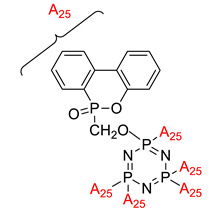	- LOI value: 34% - Additive: DOPO-TPN - Molding: Poly(ethylene terephtthalate) PET UL-94: Achieved V-0 rating	[53]
The influence of synergistic effects of hexakis (4-nitrophenoxy)cyclotriphosphazene and POE-g-MA on anti-dripping and flame retardancy of PET	Hexakis (4-nitrophenoxy)cyclotriphosphazene (HNCP) 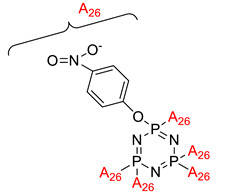	- LOI value: 28.3% - Additive used: 10 wt% - Molding: Poly(etyhylene terepththalate PET - UL-94: PET/10 wt% HNCP with 0.5 wt% POE-g-MA achieved a rating of V-0 and PET/10 wt% HNCP with 3 wt% POE-g-MA achieved rating V-0	[54]
Design of a self-healing and flame-retardant cyclotriphosphazene-based epoxy vitrimer	Cyloliner cylotriphosphazene-based epoxy resin (CTP-EP) 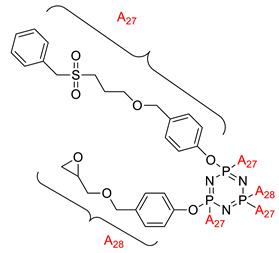	- LOI value: 30.5% - Additive used: CTP-EP/DTDA - Molding: Epoxy Resin UL-94: Achieved V-0 rating.	[55]
Synthesis and flame retardant efficacy of hexakis(3-(triethoxysilyl)propyloxy)cyclotriphosphazene/silica coatings for cotton fabrics	Hexakis(3-(triethoxysilyl)propyloxy)cyclotriphosphazene (HTPC) 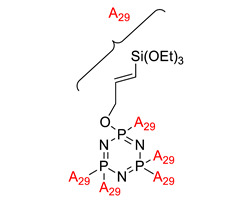	- LOI value: 27.7% - Additive used: Hexakis(3-(triethoxysilyl)propyloxy)cyclotriphosphazene (HTPC) - Molding: Cotton Fibre UL-94: Not mentioned in this study	[56]
Synthesis and characterization of thermally stable and flame retardant of hexakis(4-aminophenoxy)cyclotriphosphazene-based polyimide matrices	Hexakis(4-aminophenoxy)cyclotriphosphazene-based polyimide matrices (HACTP) 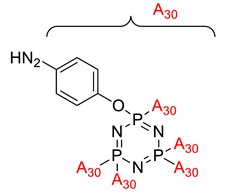	- LOI value: Range from 44.3% until 46.7%, respectively - Additive: HACTP - Molding: PI-PMDA and PI-BPDA UL-94: Achieved V-0 rating	[57]
The synthesis, curing kinetics, thermal properties, and flame retardancy of cyclotriphosphazene-containing multifunctional epoxy resin	Hexa-[4-(glycidyloxymethyl)phenoxy]-cyclotriphosphazene (HGPCP) 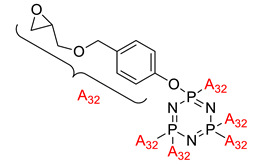	- LOI value: 32.5% and 35.4%, respectively - Additive: HGPCP/DDS and HGPCP/DDM - Molding: Epoxy Resin UL-94: Achieved V-0 rating	[58]
Hexa-[4-(glycidloxycarbonyl)phenoxy]cyclotriphosphazene chain extender for preparing high-performance flame retardant polyamide-six composites	Hexa-[4-(glycidloxycarbonyl)phenoxy]cyclotriphosphazene (CTP-EP) 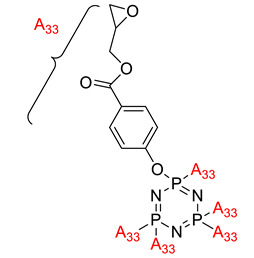	- LOI value: 31.3% - Additive: 11 wt% of ALPi/CTP-EP - Molding: Polyamide 6/aliminium diethylphpsphinate (PA6/AlPi) UL-94: Achieved V-0 rating	[59]
Synergistic effect of the intumescent flame-retardant system consisting of hexophenoxy cyclotriphosphazene and ammonium polyphosphate on methyl ethyl silicon rubber	Hexophenoxy cyclotriphosphazene (HPCP) 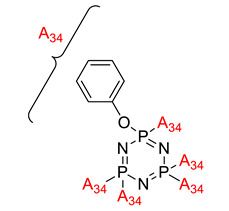	- LOI value: 30.6% - Additive: Hexophenoxy cyclotriphosphazene (HPCP) - Molding: Methyl ethyl silicone rubber (VMQ) UL-94: Achieved V-0 rating	[60]
High transmittance and environmentally friendly flame-retardant optical resin based on poly(methyl methacrylate) and cyclotriphosphazene derivatives	Ethyl p-hydroxybenzoate derivatives of cyclotriphosphazene 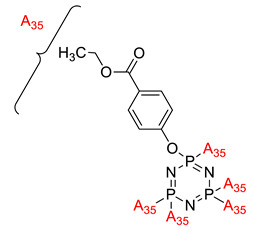 Phenol derivatives of cyclotriphosphazene 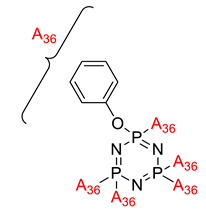	- LOI value: 26% and 22%, respectively - Additive: Two cyclotriphosphazene derivatives - Molding: High transparent optical resin based on methacrylate (PMMA) UL-94: Not mentioned in this study	[61]
Study of thermal properties of flame-retardant epoxy resin treated with hexakis[*p*-(hydroxymethyl)phenoxy] cyclotriphosphazene	Hexakis[*p*-(hydroxymethyl)phenoxy] cyclotriphosphazene (HHPCP) 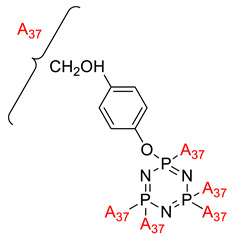	- LOI value: 26.5% - Additive: Hexakis[p-(hydroxymethyl)phenoxy] cyclotriphosphazene (HHPCP) - Molding: Epoxy Rein UL-94: Not mentioned in this study	[62]
The flame retardancy and thermal properties of poly (ethylene terephthalate)/cyclotriphosphazene modified by the montmorillonite system	Hexachlorocyclotriphosphazene mofidies by montmorillonite (HCCP-OMMT) 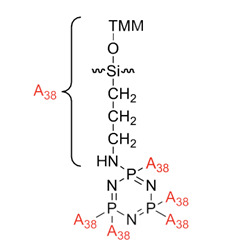	- LOI value: 31.5% - Additive: 3% of hexachlorocyclotriphosphazene mofidied by montmorillonite (HCCP-OMMT) - Molding: Poly(ethylene terephthalate) UL-94: Achieved V-0 rating	[63]
Synthesis, characterization, and utilization of novel phosphorus/nitrogen-containing flame retardant	Hexa(phosphaphenanthrene aminophenoxyl)-cyclotriphosphazene (HPAPC) 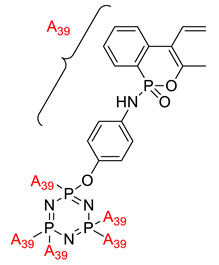	- LOI value: 34.7% - Additive: Hexa(phosphaphenanthrene aminophenoxyl)-cyclotriphosphazene (HPAPC) - Molding: Poly(lactic acid) (PLA) UL-94: Achieved V-0 rating	[25]
Effect of surface chemical modification for aluminum hypophosphite with hexa-(4-aldehyde-phenoxy)-cyclotriphosphazene on flame retardancy, water resistance, and thermal properties for polyamide 6	Hexa-(4-aldehyde-phenoxy)-cyclotriphosphazene (AHP) 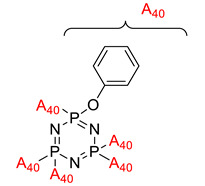	- LOI value: 27.6% - Additive: Hexa-(4-aldehyde-phenoxy)-cyclotriphosphazene (AHP) - Molding: Polyamide 6 (PA6) - UL-94: Achieved V-0 rating	[64]
Synthesis of tris(phenoxy)triflorocyclotriphosphazene and study of its effect on the flammable, thermal, optical, and mechanical properties of bisphenol-A polycarbonate	Tris(phenoxy)trifluorocyclotriphosphazene (TCTP) 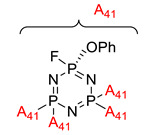	- LOI value: 40% - Additive: Tris(phenoxy)trifluorocyclotriphosphazene (TCTP) - Molding: Polycarbonate - UL-94: Achieved V-0 rating	[65]
Effect of trisilanolphenyl-POSS on rheological, mechanical, and flame retardancy properties of the poly(ethylene terephthalate)/cyclotriphosphazene system	Hexakis (para-alloxyphenoxy) cyclotriphosphazene (PACP) 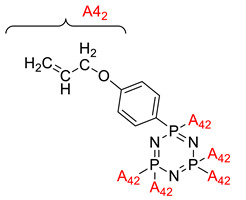	- LOI value: Not mentioned in this study - Additive: Hexakis (para-alloxyphenoxy) cyclotriphosphazene (PACP) - Molding: Poly(ethylene terepthtalate) (PET) UL-94: Achieved V-0 rating	[66]
Preparation and properties of novel inherent flame-retardant cyclotriphophazene containing epoxy resin	Bis-(4-hydroxyphenylsulfonylphenoxy)tereohenoxycyclotriphosphazene (HSPPZ) 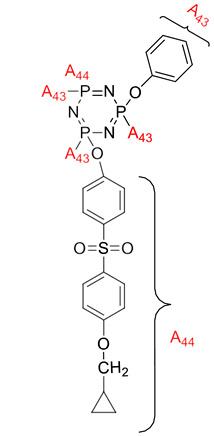	- LOI value: Not mentioned in this study - Additive: Bis-(4-hydroxyphenylsulfonylphenoxy) tereohenoxycyclotriphosphazene (HSPPZ) - Molding: Epoxy Resin UL-94: Achieved V-0 rating	[67]

**Table 2:** Synthesis of the hexasubstituted cyclotriphosphazene compound with several types of linking units.

Title of Journal	Types of Linking Unit	Description	Ref.
Synthesis of new star-shaped liquid crystalline cyclotriphosphazene derivatives with flame-retardant bearing amide-azo and azo-azo linking units.	Amide azo and azo-azo linking units	- This study aimed to synthesize two series of new hexasubstituted cyclotriphosphazene containing two types of linking units: amide-azo and azo-azo. - The homologues of the same series contain different terminal substituents such as heptyl, nonyl, decyl, dodecyl, tetradecyl, hydroxyl, carboxyl, chloro, nitro, and amino. - The flame retardancy of synthesized compounds is measured using the Limiting Oxygen Index. - From the result obtained, compounds containing heptyl have a higher LOI value compared with other compounds. The LOI values for alkylated compounds decrease as the aliphatic chain length increases. This study also compared the flame retardancy properties of two compounds containing two different linking units. The compound containing amide-azo was reported to have a high LOI value. The phenomenon indicates the attribute to the electron withdrawing properties of amide moiety.	[73]
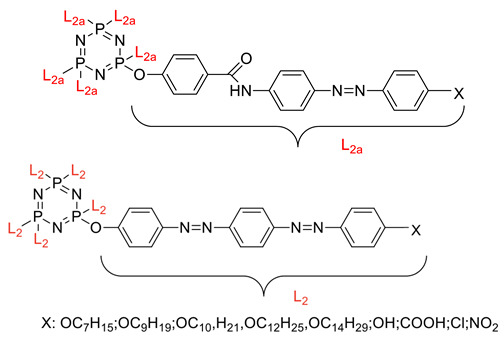			
Synthesis of novel liquid crystalline and flame-retardant molecules based on six armed cyclotriphosphazene cores containing Schiff base and amide linking units.	Schiff base and amide linking units	- This study aimed to synthesize hexa substituted cyclotriphosphazene containing Schiff base and amide linking units, characterization, and chemical testing (flame retardancy using LOI value) were also measured. - From the result obtained, the LOI value indicating the flame retardancy properties of the compound showed that the compound with the nitro group had a high LOI value due to the electron withdrawing group that enhances the synergistic effect of P-N bonds. - This study also reported that cyclotriphosphazene compounds could enhance the flame retardancy of polyester resin. The LOI value of polyester resin increased from 22.53% to 24.71%. Schiff base linking unit was found to enhance these properties due to char formation in the condensed phase. - Other than that, the amide linking unit also contributed to the increase of LOI value. This is because the electron withdrawing of the amide bond enhances the flammability of these compounds.	[74]
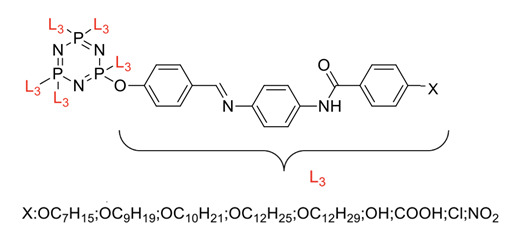			
Synthesis, characterization, and mesophase transition of Hexasubstituted cyclotriphosphazene molecules with Schiff base and Azo linking units and determination of their flame-retardant properties.	Schiff base and azo linking units	- This study aimed to synthesis hexasubstituted cyclotriphosphazene containing Schiff base and Azo linking units. The characterization and chemical testing (flame Retardancy using LOI value) were also measured. - From the result obtained, the LOI valued increased when incorporated with hexasubstituted cyclotriphosphazene. The LOI value of the compound containing the nitro group was recorded as the highest, which was 27.90%. This is due to the nitro group’s electron withdrawing, which releases the electron from their resonance effects to their corresponding P-N bonds. As a result, the P-N synergistic effect was enhanced, and they exhibited both the condensed and gas phase action, which caused the compound to have a high LOI value.	[75,76]
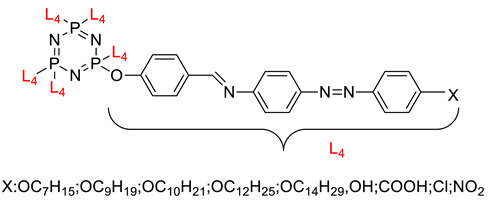			

**Table 3:** Cyclotriphosphazene compound bearing oxime ether and ester as the side group.

Compound	Name
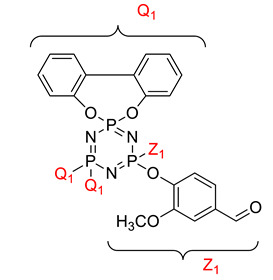	2,2-Bis(4-formyl-2-methoxyphenoxy)-4,4,6,6-bis[spiro(2′,2″- dioxy-1′,1″-biphenylyl)] cyclotriphosphazene
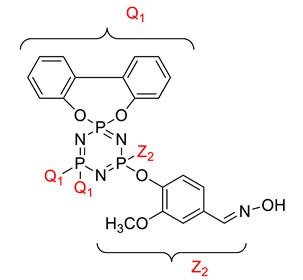	2,2-di[(4-(hydroxyimino)-2-methoxy)phenoxy]- 4,4,6,6-bis[spiro(2′,2″-dioxy-1′1″-biphenylyl)]cyclotriphosphazene
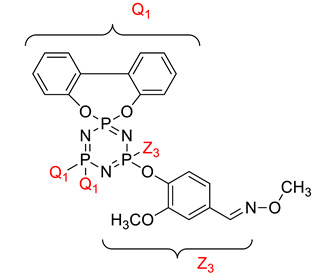	2,2-di[{(4-(methyloxy)imino)-2-methoxy} phenoxy]-4,4,6,6-bis[spiro(2′2″-dioxy-1′,1″-biphenylyl)] cyclotriphosphazene
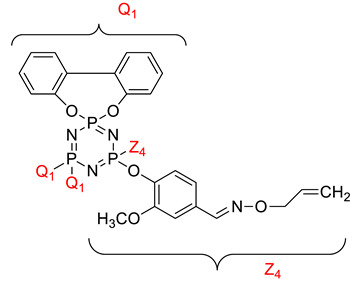	2,2-di[{(4-(allyloxy)imino)-2-methoxy} phenoxy]-4,4,6,6-bis[spiro(2′,2″-dioxy-1′,1″-biphenylyl)] cyclotriphosphazene
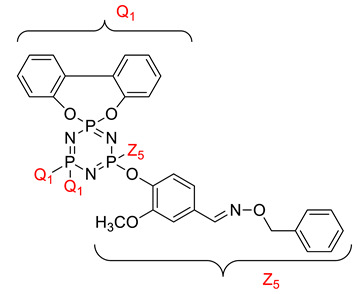	2,2-di[{(4-(benzyloxy)imino)-2-methoxy} phenoxy]-4,4,6,6-bis[spiro(2′,2″-dioxy-1′,1″-biphenylyl)] cyclotriphosphazene
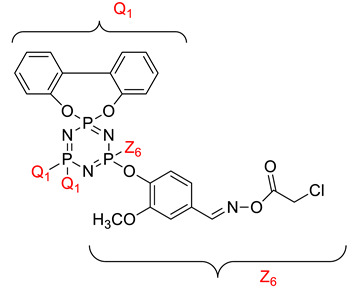	2,2-di[{(4-(chloroacetyloxy)imino)-2-methoxy} phenoxy]-4,4,6,6-bis[spiro(2′,2″-dioxy-1′,1″-biphenylyl)] cyclotriphosphazene
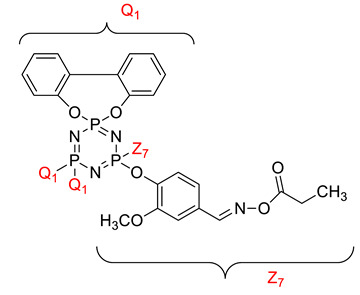	2,2-di[{(4-(propanoyloxy)imino)-2-methoxy} phenoxy]-4,4,6,6-bis[spiro(2′,2″-dioxy-1′,1″-biphenylyl)] cyclotriphosphazene
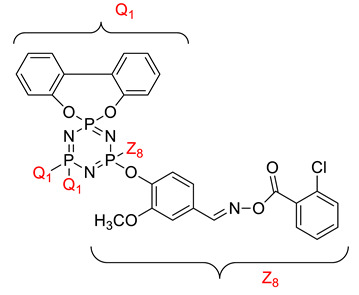	2,2-di[{(4-(o-chlorobenzoyloxy)imino)-2- methoxy}phenoxy]-4,4,6,6-bis[spiro(2′,2″-dioxy-1′,1″-biphenylyl)] cyclotriphosphaz
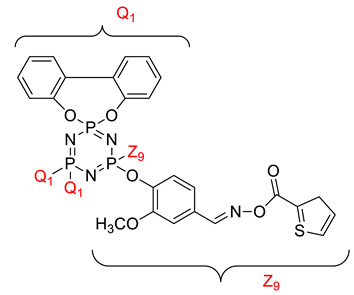	2,2-di[{(4-(thiophene-2-carbonyloxy)imino)-2- methoxy}phenoxy]-4,4,6,6-bis[spiro(2′,2″-dioxy-1′,1″-biphenylyl)] cyclotriphosphazene

**Table 4:** Reported study of cyclotriphosphazene and their dielectric properties.

Compound Name	Dielectric Constant	Dielectric Loss	Ref.
2,2,4,4-tetra(4′-oxy-substituted-chalcone)- 6,6-diphenyl cyclotriphosphazene derivatives 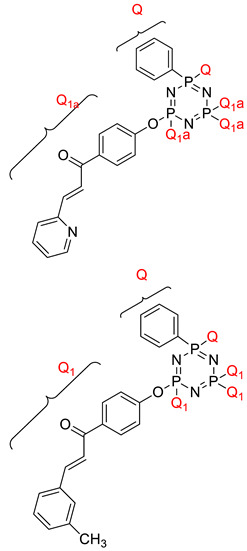	- Dielectric constants decreased with the increasing frequency, while they remained constant at high frequencies. **Structural Reasons**: This can be thought to be a polarization effect. Polarization occurs since the effect of dipole increases the frequencies. - Dielectric constant hexasubstituted cyclotrphosphazene compound containing Cl in ortho position was found as higher compared with the synthesized compound of hexasubstituted cyclotriphosphazene containing F atom in ortho position. **Structural Reasons**: The chlorine atom in the structure increased the polarity, and hence the dipole moment increased. - Dielectric constant of the synthesis compound containing pyridine was recorded to be the highest. **Structural Reasons**: This is due to the presence of the hetero atom in a ring, which can contribute to higher polarization.	Dielectric loss values of the compounds decreased along with the increasing frequency and remained unchanged after some point.	[4]
Mono(4-fluorobenzyl)cyclotriphosphazene derivatives with (dimethylamino)ethoxy and (dimethylamino)propoxy chains. 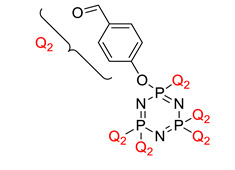	- Sharp decrease of the dielectric constant in the low region of frequency. **Structural Reasons**: This is due to the relaxation process of the diffusion ion. - Compounds containing ethyl groups as an alkyl chain had a higher dielectric constant than those containing methyl groups. **Structural Reasons**: Increasing the side chain resulted in a deteriorated charge transport and unfavorable intermolecular interactions, leading to a decreasing dielectric constant.	-	[91,92,93]
Hexasubstitued cyclotriphosphazene compounds containing chalcone derivatives 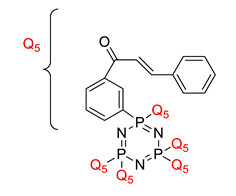 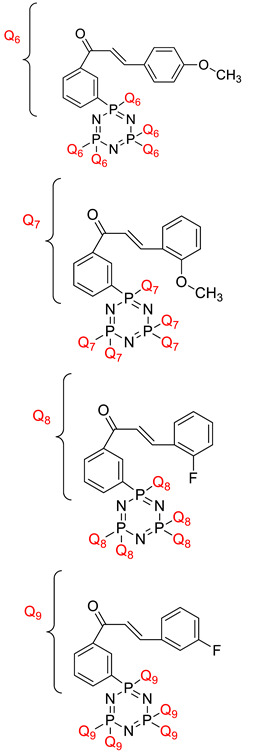	- Dielectric constant decreased with increasing frequency. **Structural Reasons:** This is because the atom in the compound was consistent with the direct charge of the alternating electric field (AEF) at low frequencies that reduce the dielectric constant value at high frequencies.	- Dielectric loss of the sample decreased with increasing frequency. **Structural Reasons**: Interfacial dipoles had less time to orient themselves in the direction of the alternate field. As a result, the low-frequency region is attributed to the contribution of charge accumulation at the interface.	[94]
Cyclotri(trifluoroethoxy, acryloyloxy-ethyleneoxy)phosphazene 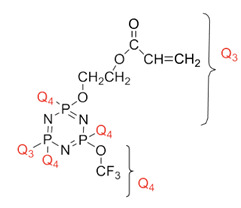	- Dielectric constant increased with increasing temperature and with decreasing frequency. **Structural Reasons:** Due to the rigidity of the polymer matrix.	- Dielectric loss increased with increasing temperature and with decreasing frequency. **Structural Reasons**: Due to the viscosity of the PNF polymer decrease, which favors dipole orientation.	[95]

**Table 5:** Structural contribution of the enhancement of flame-retardant and dielectric properties.

Addictive	Polymer Matrix	Properties Studies	Finding(s)	Ref.
Hexa(aminophenyl)cyclotriphosphazene	Cyanate ester (CE)	Thermal properties Dielectric properties	Limiting oxygen index (LOI) increased. **Structural reasons**: CPA contains phosphorus, and nitrogen compounds exhibit a good flame retardancy by forming phosphorus char that will act as a protecting layer of heat transfer that will reduce combustible gas production. Nitrogen in CPA will enhance the formation of phosphorus char and a nitrogen compound, which is also efficient in preventing oxygen from burning materials [105]. Dielectric loss and dielectric constant of CPA/CE lower than neat CE. **Structural reasons**: CPA has bulky sextet moiety with flexible structure; hence incorporated CE composites with CPA contribute to low dielectric properties.	[96]
Cyclotriphosphazene reinforced polybenzoxazine (PBZ)	EP/PZT	Flame-retardant properties Dielectric properties (Temperature and Volume)	Limiting oxygen index (LOI) increased. **Structural reasons**: Incorporated phosphorus and nitrogen-containing phosphazene fiber into the FBZ/EP matrix contributed to flame retardancy since the phosphorus can act in a condensed phase to promote char formation, shielding the polymer from heat and air. Dielectric loss and dielectric constant increased with decreasing the temperature. **Structural reasons**: It can be ascertained that the composite samples exhibit stable dielectric behavior with regard to variation in temperature. Dielectric constant decreased when the volume of the loaded composite increases. **Structural reasons:** The dielectric of polymer was determined by the three factors in this study: volume, charge distribution, and statically thermal motion of the polar group. The reduction in the dielectric constant may be explained due to the enhancement of the free volume and, to a certain extent, due to the influencing effect of PZT fiber.	[98]
Hexa(aminophenyl)cyclotriphosphazene (PZA)	PZI matrix	Flame-retardant properties Dielectric constant	Limiting oxygen index (LOI) of PZA/PZI matrix was higher than neat PZI. **Structural Reasons:** Due to the nitrogen and phosphorus in skeletal moiety [105]. In addition, the network structure of PZI matrix also offered an alternative to phosphazene cores. Low dielectric constant obtained from this compound. **Structural Reasons:** Introduction of such porous materials creates a free volume and contributes to obtaining materials that have a low dielectric constant.	[103]
BICP	Epoxy Resin	Fire retardant Dielectric constant	Limiting oxygen index (LOI) of curing EP with BICP improved. **Structural Reasons:** These organophosphorus compounds facilitated the formation of more residual chars with better thermo-oxidative stability. Dielectric constant and loss of EP/BICP thermosets were lower in comparison with those of the EP/BIM10 thermoset over a wide frequency range. **Structural Reasons**: Increased free volume of the EP matrix due to the incorporation of a bulky, rigid BICP molecule might be responsible for the decreased dielectric constant of EP/BICP thermosets. Moreover, in the case of thermosets with the homogeneous network, the dielectric properties of polymers generally depend on the orientation and relaxation of dipoles which are closely related to the movement of polymer-chain segments [106].	[104]
HSSPZ	Epoxy Resin	Flame-retardant Electrical properties	The UL-94 achieved a V-0 rating. **Structural reasons**: This is attributed to the high nitrogen and phosphorus content in the resins. These are believed to have a synergistic effect during burning and yield the higher char amount that can retard the flammability of the materials. The resistivity of the compound increased. **Structural Reasons:** Inorganic ring of alternating phosphorus and nitrogen atoms of cyclotriphosphazene skeleton in the molecular backbone. The skeleton of cyclotriphosphazene is not a conjugated structure [107], and no electron can be supplied. Thus, the addition of CPEP could retain the electrical resistance of EP.	[67]

## Data Availability

Not applicable.
